# Thermodynamic Simulation of Environmental and Population Protection by Utilization of Technogenic Tailings of Enrichment

**DOI:** 10.3390/ma15196980

**Published:** 2022-10-08

**Authors:** Olga Kolesnikova, Samal Syrlybekkyzy, Roman Fediuk, Almas Yerzhanov, Rashid Nadirov, Akmaral Utelbayeva, Aktolkyn Agabekova, Marina Latypova, Larissa Chepelyan, Irina Volokitina, Nikolai Ivanovich Vatin, Alexandr Kolesnikov, Mugahed Amran

**Affiliations:** 1 Department of Science of Production and Innovation, Department of Life Safety and Environmental Protection, M. Auezov South Kazakhstan University, Shymkent 160012, Kazakhstan; 2 Department of Ecology and Geology, Sh. Yesenov Caspian University of Technology and Engineering, Aktau 130002, Kazakhstan; 3 Polytechnic Institute, Far Eastern Federal University, 690922 Vladivostok, Russia; 4 Peter the Great St. Petersburg Polytechnic University, 195251 St. Petersburg, Russia; 5 Department of Metal Forming, Department of Economics and Business, Karaganda Industrial University, Temirtau 101400, Kazakhstan; 6 Department of General and Inorganic Chemistry, Al-Farabi Kazakh National University, Almaty 050040, Kazakhstan; 7 Laboratory of Mechanochemical Processes and Combustion Problems, Institute of Combustion Problems, Almaty 050012, Kazakhstan; 8 Department of Electrical Engineering, H. A. Yassavi International Kazakh-Turkish University, Turkestan 161200, Kazakhstan; 9 Department of Metallurgy and Mining Rudny Industrial Institute, Rudny 111500, Kazakhstan; 10 Department of Civil Engineering, College of Engineering, Prince Sattam Bin Abdulaziz University, Alkharj 16273, Saudi Arabia; 11 Department of Civil Engineering, Faculty of Engineering and IT, Amran University, Amran 9677, Yemen

**Keywords:** thermodynamics simulation, environmental and population protection, recycling, waste, raw materials, zinc ferrite, cement clinker minerals, zinc sublimations

## Abstract

During mining, only 4–8% is converted to final products, and the rest is accumulated in landfills. There is a lack of research on the study of various patterns and mechanisms of the formation of cement clinker minerals during the simultaneous distillation of zinc. This paper presents studies of thermodynamic stimulation of environmental and population protection by utilization of technogenic enrichment waste as secondary raw materials for clinker production and zinc extraction. In particular, a comparison of the Gibbs energy (ΔG) of clinker formation under standard chemical equations and under non-standard chemical equations is given. According to the results of the study, using thermodynamic simulation, the temperature intervals of mineral formation, the dependence of the Gibbs energy on temperature (ΔG_T_°), and the approximation equations were found; it was established that the presence of zinc ferrite contributes to the intensification of the formation of clinker minerals and the extraction of Zn to gas.

## 1. Introduction

**Figure 1:**
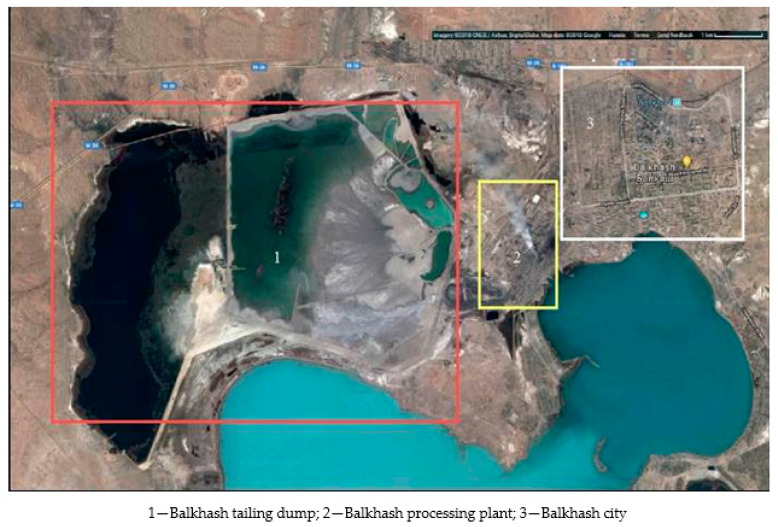
A photo from space of a general view of the Balkhash tailings dump (1), an enrichment plant (2) and the city of Balkhash (3).

According to various data, more than 1.53 billion tons of solid minerals are extracted annually in the Republic of Kazakhstan, of which the third part (534 million tons) is for metallurgy [1,2]. However, not much more than 4–8% of this amount is converted into final products, and the rest is exported to landfills in the form of overburden rocks, enrichment tailings (Figure 1) and metallurgical slags; more and more is accumulating and occupying significant territories. Moreover, most of the waste (57.6%) falls on non-ferrous and ferrous metallurgy (38.9 and 23.7%, respectively). In the coal industry, waste reached 1.8 billion tons, about 12% of the total amount in the Republic of Kazakhstan [1]; in the energy sector (ash slag), 1.2%; and in the phosphorus industry, 2.3%

The level of involvement of man-fabricated industrial waste as secondary raw materials is frankly low, namely: in the context of ferrous metallurgy, 3.3%; in the context of the coal industry, 5.2%; and in the context of non-ferrous metallurgy, 5.6%, which is a very low indicator in relation to the developed economies of the world.

Currently, the modern world requires resources, which in the future will have a separate position in the development of the world global economy [1,2] and the economy of Kazakhstan [3,4]. However, at the same time it is necessary to critically review the organization of raw material industries, namely to reconsider the approaches to the management of natural resources. It is essential to implement comprehensive information technology services for natural and anthropogenic raw materials. The requirements for energy and resource efficiency of industrial enterprises in the chemical, energy, mining, and metallurgical sectors, as well as their environmental friendliness and efficiency, should be significantly improved [5,6,7,8,9,10,11,12,13,14].

At present, due to the decrease in valuable metals in ores and the increasing amount of hard-to-enrich raw materials [14,15,16,17,18,19,20,21,22] it is economically feasible and necessary to comprehensively process both poor, non-standard, and hard-to-access mineral raw materials and technogenic ones [6,7,8,9,10,11,12,13,14,15,16,17,18,19,20,21,22,23]. These include those in dumps, tailings of Kazakhstan, Uzbekistan, Russia, Ukraine, Finland, Poland, Romania, Argentina, and Canada.

The main resources in the production of binders were previously provided by traditional mineral raw materials. The new economic realities are changing this approach qualitatively, and industrial wastes come forward as secondary raw materials [6,7,8,9,10,11,12,13,14,15,16,17,18,19,20,21,22]. The cost of such raw materials is significantly less, and the conditions for processing are often simple. These features of economic development insistently require the qualitative study of all kinds of accumulated and unused waste. Such wastes are ones from the mining, processing, and metallurgical industries such as tailings from the Balkhash Processing Plant (BPP) in Kazakhstan [6,7,8,23,24]. They include a number of useful compounds, in particular, the oxides of silicon, aluminum, and iron. They are essential for the production of cement clinker, as well as non-ferrous metal compounds such as zinc, which is one of the valuable metals in the metallurgical industry [6,8,14,15,19].

The scientific community has repeatedly conducted various experiments with the influence of non-ferrous metals on the properties and processes in the production of cement clinker. In particular, Zaid et al. [25] conducted a study on the effect of zinc oxide on the phase composition of clinker and cement properties. During the research at the Krakow branch of the Institute of Ceramics and Building Materials, a series of clinkers with different zinc content was produced in a semi-industrial rotary kiln. According to the research results, it was shown that with a content of up to 1.2% zinc in clinker, the properties of cement do not deteriorate. A high zinc content (2.13%) leads to a change in the properties of cement due to the formation of ZnO in the clinker, which can contribute to the process of slowing down the setting time and inhibit the hydration of alite.

Guineis et al. [26] determined the threshold limits for Zn when introduced into a standard Portland cement clinker. These threshold limits were studied in laboratory synthesized clinkers using X-ray and scanning electron microscopy. During the research, it was established that the mineralogical composition of the synthesized clinker was close to the standard Portland cement clinker, and the reactivity of cements fabricated using clinkers within the threshold limits (1% Zn) was accompanied by calorimetry and measurements of the compressive strength of cement paste. The results of the studies showed that the alloyed cements were at least as reactive as the reference cement.

Perez-Bravo et al. [27] studied the effect of zinc oxide on the clinker formation process. Clinkers with the addition of 1% zinc oxide were fired at a temperature of 1300 °C. The addition of insignificant amounts of zinc oxide to the raw materials contributed to a certain decrease in temperature due to the formation of a liquid phase during the firing process.

Having considered the scientific experience of a number of researchers from the world scientific community on the use of zinc-containing raw materials in the production of cement clinker, it was decided to conduct thermodynamic studies using the Gibbs energy calculation (ΔG_T_°). In particular, the process of simultaneous synthesis of the main cement clinker minerals formation (Ca_2_SiO_4_–C_2_S, alite; Ca_3_SiO_5_–C_3_S, belite; Ca_3_Al_2_O_6_–C_3_A, tricalcium aluminate; and Ca_4_Al_2_Fe_2_O_10_–C_4_AF, four-calcium aluminoferrite) and zinc sublimations depending on temperature from mineral (limestone) and technogenic (containing zinc ferrite, ZnFe_2_O_4_ [28]) in in the form of tailings from the enrichment of non-ferrous metal ores. However, there is a lack of research on the study of various patterns of the formation of cement clinker minerals during the simultaneous distillation of zinc.

Thus, the studies aimed at reducing the energy costs and specific consumption of raw materials, engaging technogenic raw materials in the production cycle as secondary raw materials while reducing the environmental impact through waste recycling are relevant, novel, and require a comprehensive qualitative approach for their further development and implementation into production [6,8,17,25,26,27,28,29,30,31,32].

## 2. Materials and Methods

Limestone of the Mynaral deposit (Mynaral settlement, Kazakhstan) was considered as a mineral raw material. The limestone deposit is located 20 km northwest of the Mynaral railway station in the Moyinkum district of Zhambyl region (Kazakhstan). It was opened in 1958. The area of the deposit belongs to the Balkhash trough, made mainly by formations of the Hercynian time, and located at the junction of the Chu-Balkhash geoanticlinal and Jungar–Balkhash geosynclinal zones. The boundary between them can be traced along the large faults of the Akkyrminsky system of the North direction, separating the Lower Paleozoic uplift from the Devonian Karakamyss brachysyncline in the southwest. The site of the deposit is confined to the southern wing of the synclinal structure, is composed of effusive-pyroclastic detachments of the upper suite of the lower-Middle Devonian. The covers of fluidic and massive felsite porphyries alternating with the horizons of lavobreccia and bubbly lavas in the eastern part of the area were broken by a large multiphase vent apparatus. Interplastic deposits, stocks, and dykes are also intensively developed and have different bodies of acidic composition, such as felsites, plagioporphyres, and quartz porphyries. The cover and vent formations in the north of the site with angular and azimuthal disagreement are overlain by red-colored Middle Devonian tuff conglomerates and tuff sandstones. The general strike of the rocks is northwest, falling to the north and northeast at angles of 10–20°. The multiphase volcanic apparatus of the central type has a rounded shape in plan and mushroom-shaped in section, framed by secant extrusive bodies of acid composition and powerful fields of post-volcanic rock changes up to secondary quartzites. Late dikes of plagioporphyres, diabases and diabase porphyrites are widely developed in the form of belts of sublatitudinal, submeridional and northwestern directions, with a node of mutual intersection in the exocontacts of the volcanic apparatus. The sublatitudinal and northwestern faults of the Akkerman zone system, a number of northeastern (up to sublatitudinal) disturbances, and hidden faults of the meridional strike are clearly manifested. In the south of the area, the sublatitudinal Mynaral fault is traced, accompanied by dikes of plagioporphyres and diabase porphyries, zones of schisms and quartz-sericite changes. The explored three ore deposits are confined to the site of the southeastern wedging of the fluidic felsite porphyry horizon at its junction with the secant body of plagioporphyres and the contact of the volcanic vent (ore site II). The fall of the horizon on the SV at angles of 20–40°, the stretch is northwest, and the power is 250 m. Limestone of the deposit is represented by the following chemical compounds by mass%: SiO_2_, 5.47; A_2_O_3_, 1.73; Fe_2_O_3_, 0.69; CaO, 48.24; MgO, 0.71; ppp, 37.63; and other, 5.53 [33]. Moreover, tailings from the enrichment of the Balkhash processing plant are technogenic raw materials (Balkhash, Kazakhstan). The tailings of enrichment are already crushed, homogeneous, and often fractionated raw materials. The Balkhash processing plant, which owns the tailings dump, enriches non-ferrous metal ores of the Kounrad and Sayak deposits. An average of 33.7 thousand tons of Kounrad and 9.5 thousand tons of Sayak ores are processed at the factory per day. The enrichment waste is stored in a tailings dump. The tailings dump of the Balkhash Processing Plant is of particular great interest since it is one of the oldest in the industry and one of the largest tailings dumps in Kazakhstan. In addition, it is not located in a simple arid zone, which in itself already significantly increases the impact of the tailings dump on the environment [34], it is located very close to Lake Balkhash which has a large, even huge, economic purpose. Chemical (average) composition of tailings from the enrichment of the Balkhash processing plant according to various scientists [1,5,6,19,34,35]: represented by the following compounds by mass%: SiO_2_, 52.20; A_2_O_3_, 13.40; Fe_2_O_3_, 8.13; CaO, 8.25; MgO, 0.55; Zn, 1.37; Pb, 0.44; Cu, 0.1; loss on ignition, 11.26; other (Na, S, K, and Ti), 4.43

Thermodynamic modeling was carried out on the basis of the chemical and mineral composition of limestone and tailings from enrichment using the program “Thermodynamics” (Moscow, Russia) [36] and the software package “HSC Chemistry 6” [37], developed by the metallurgical company Outotec (Helsinki, Finland). The software used in this work is based on the ideology of the European consortium Scientific Group Thermodata Europe (SGTE), which develops, maintains, and distributes quality databases. The SGTE structure is represented by the dedicated scientific centers in Germany, Canada, France, Sweden, the UK, and the USA. The software’s database contains information on 22,000 individual substances [37].

To calculate the thermodynamic functions characterizing an individual substance, we used the standard values of enthalpy *H*_298_, entropy *S*_298_, and polynomial coefficients *A*, *B*, *C*, and *D* stored in the database, from which the molar heat capacity was calculated at an arbitrarily specified temperature *T* [36,37].

The enthalpy of an individual substance at a temperature *T*, which differs from the standard one equal to 298 K, was calculated by the formula:

(1) $$H_{T}=H_{298}+\int_{298}^{T}C_{p}dT+\sum H_{F},$$

where *H*_298_ is the enthalpy value of a given substance under standard conditions; *C_p_* is the molar heat capacity; and ∑*H_F_* is the enthalpy of phase transitions (polymorphic transformations, melting, and evaporation).

The entropy is defined as:

(2) $$S_{T}=S_{298}+\int_{298}^{T}\frac{C_{p}}{T}dT+\frac{\sum H_{F}}{T},$$

where *S*_298_ is the value of the entropy of a given substance under standard conditions; *C_p_* is the molar heat capacity; and $\frac{\sum H_{F}}{T}$ is the entropy of phase transitions (polymorphic transformations, melting, and evaporation) [36,37].

The Gibbs energy (ΔG_T_°) in this software package is calculated using the following formula:

*G*_*T*_ = *H*_*T*_ − *TS*_*T*_. (3)

The main difficulty in calculating the thermodynamic characteristics of a substance at an arbitrary temperature is the calculation of temperature corrections in the form of integrals in Equations (1) and (2) [36,37].

## 3. Results

During the simulation using thermodynamics, Gibbs energy values (ΔG_T_°) were obtained as a function of temperature using software packages. Under the conditions of modeling the process of utilization of tailings from the enrichment of non-ferrous metal ores (containing a compound in the form of ZnFe_2_O_4_) with the formation of mineral phases of clinker (Ca_2_SiO_4_, Ca_3_SiO_5_, Ca_3_Al_2_O_6_, and Ca_4_Al_2_Fe_2_O_10_) and simultaneous distillation of zinc (Zn) to sublimations (gaseous state) at the studied temperature range of 600–1600 °C with use as a natural mineral raw material, a series of thermochemical equations were thermodynamically investigated for limestone containing CaCO_3_. First of all, a number of well-known chemical Equations (4)–(7) for the formation of mineral phases of a simulated sinter clinker were considered [21,23,32,38,39,40,41]:

2CaCO_3_ + SiO_2_ → Ca_2_SiO_4_ + 2CO_2_ (4)

3CaCO_3_ + SiO_2_ → Ca_3_SiO_5_ + 3CO_2_ (5)

3CaCO_3_ + Al_2_O_3_ → Ca_3_Al_2_O_6_ + 3CO_2_ (6)

4CaCO_3_ + Al_2_O_3_ + Fe_2_O_3_ → Ca_4_Al_2_Fe_2_O_10_ + 4CO_2_ (7)

In the course of the simulation, based on calculations of the results of a number of thermochemical equations, based on the dependence (ΔG_T_°) on temperature, the values of ΔG_T_° were obtained by calculation, indicating the possibility of the process of mineral phase formation of the simulated sinter and the flow of a number of chemical equations subjected to Researches (4)–(7) in Table 1.

A number of investigated thermochemical equations, represented by Equations (8)–(11) are based on the interaction of zinc ferrite (ZnFe_2_O_4_) with CaCO_3_ and Al_2_O_3_ with the formation of mineral phase formation of the simulated sinter clinker:

ZnFe_2_O_4_ + 2CaCO_3_ + SiO_2_→Zn + Ca_2_SiO_4_ + Fe_2_O_3_ + 2CO_2_ + 0.5O_2_ (8)

ZnFe_2_O_4_ + 3CaCO_3_ + SiO_2_→Zn + Ca_3_SiO_5_ + Fe_2_O_3_ + 3CO_2_ + 0.5O_2_ (9)

ZnFe_2_O_4_ + 3CaCO_3_ + Al_2_O_3_→Zn + Ca_3_Al_2_O_6_ + Fe_2_O_3_ + 3CO_2_ + 0.5O_2_ (10)

ZnFe_2_O_4_ + 4CaCO_3_ + Al_2_O_3_→Zn + Ca_4_Al_2_Fe_2_O_10_ + 4CO_2_ + 0.5O_2_ (11)

**Figure 2:**
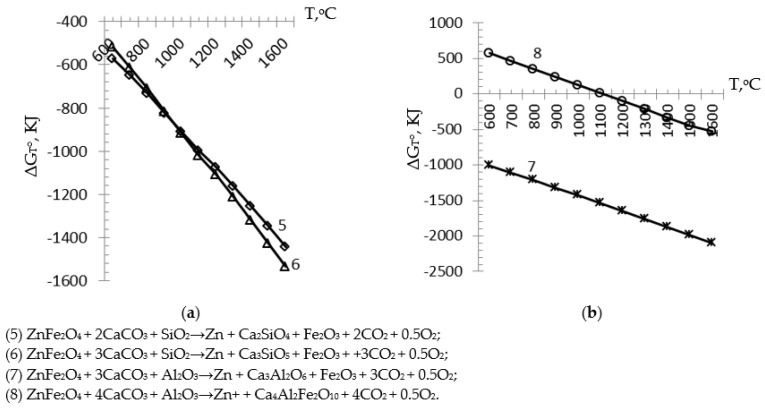
Temperature effect on the Gibbs energy of the unconventional reaction with zinc distillation and formation of Ca_2_SiO_4_, Ca_3_SiO_5_ (**a**), и Ca_3_Al_2_O_6_, and Ca_4_Al_2_Fe_2_O_10_ (**b**).

From the obtained results of calculating the Gibbs energy of thermochemical Equations (8)–(11), depending on the studied temperature interval, graphical dependences of the possible mineralogical phase formation of the simulated sinter clinker with Zn distillation into the gas-forming phase in the presence of zinc ferrite were constructed. Graphs of the dependence of a number of chemical equations are shown in Figure 2a,b.

Based on the obtained data on the simulation of a number of thermochemical equations of mineralogical phase formation of the simulated sinter clinker, approximation equations were found describing the individual chemical equations of series I (Equations (4)–(7) and II (Equations (8)–(11)) depending on T (°C) with the value of approximation reliability (R^2^):

For Equation (4) with the creation of C_2_S—ΔG_T_° = −27.724T − 44.331; R^2^ = 0.9383; (12)

For Equation (5) with the creation of C_3_S—ΔG_T_° = −42.168T + 21.3; R^2^ = 0.9712; (13)

For Equation (6) with the creation of C_3_A—ΔG_T_° = −44.434T + 123.88; R^2^ = 0.9936; (14)

For Equation (7) with the creation of C_4_AF—ΔG_T_° = −50.105T + 118.18; R^2^ = 0.9921; (15)

For Equation (8) with the creation of C_2_S—ΔG_T_° = −86.876T − 475.87; R^2^ = 0.9995; (16)

For Equation (9) with the creation of C_3_S—ΔG_T_° = −101.33T − 410.19; R^2^ = 0.9997; (17)

For Equation (10) with the creation of C_3_A—ΔGT° = −108.94T − 892.05; R^2^ = 0.9997; (18)

For Equation (11) with the creation of C_4_AF—ΔG_T_° = −112.35T + 688.24; R^2^ = 0.9994. (19)

From the obtained results of calculating the ΔG_T_° of a number of thermochemical Equations (8)–(11), depending on the temperature interval under study, graphical dependences of the possible mineralogical phase formation of the simulated sinter clinker with simultaneous transfer of Zn to gas in the presence of zinc ferrite were constructed. Graphs of the dependence of a number of chemical Equations (8)–(11) are shown in Figure 2a,b.

## 4. Discussion

From the obtained results of calculating the ΔG_T_° in Equations (4) and (5) in Table 1, it follows that the known chemical equations of mineralogical phase formation of the modeled sinter clinker have negative values of ΔG_T_° in the entire temperature range under study, which indicates their almost complete implementation and flow under these study conditions with the formation of minerals C_2_S and C_3_S.

Thus, during the formation of the C_2_S mineral in the studied temperature range, the Gibbs energy (G_T_°) has values ranging from −50.2 to −340.5 kJ at temperatures of 600 and 1600 °C, respectively, and is described by the following approximation equation ΔG_T_° = −27.724T − 44.331 with an approximation confidence value (R^2^) equal to 0.9383. The given calculated values of the G_T_° of chemical Equation (5) in comparison with chemical Equation (4) at the beginning of the studied temperature range has a positive value of ΔG_T_^o^, which is 2.3 kJ at a temperature of 600 °C, which in accordance with thermodynamic laws has the basis that under these temperature and stoichiometric conditions, Equation (5) is not possible to flow. A certain temperature of the beginning of the flow of Equation (5) is a value equal to 610 °C, that is, with a negative value of ΔG_T_°. Accordingly, the possibility of reacting Equation (5) with the subsequent formation of the mineral, C_3_S, is realistically possible in the temperature range of 605.2–1600 °C, respectively, with a ΔG_T_° from −0.006 to −432.5 kJ (Table 1) and is described by the approximation equation ΔG_T_° = −42.168T + 21.3 with an approximation confidence value (R^2^) equal to 0.9712. At the same time, the Gibbs energy of Equations (4) and (5) form a peak in the research temperature range of 1100–1200 °C, which has the possibility of explaining these peaks by polymorphism of silica. Calculated in the course of thermodynamic simulation of the Gibbs energy, Equations (6) and (7) in Table 1 have almost similar values of ΔG_T_° from temperature and have almost similar positive values of ΔG_T_° at 600 °C, 89 and 83.9 kJ, respectively. The present dependences of the values of ΔG_T_° show their limited reactivity at the initial temperature of the conducted studies. Thus, the ΔG_T_^o^ of Equation (6) with the possibility of mineral formation Ca_3_Al_2_O_6_ has a negative value at 783.58 °C and is −0.001 kJ, reaching a more negative value (−334.4 kJ) at 1600 °C and is described by the following approximation equation ΔG_T_^o^ = −44.434T + 123.88 with an approximation confidence value (R^2^) equal to 0.9936. The possible mineral formation of ΔG_T_° is Ca_4_Al_2_Fe_2_O_10_. This is shown in Table 1 in accordance with Equation (7). The found values of ΔG_T_^o^ of Equation (7) begin to become negative at 746 °C and at the same time the value is −0.528 kJ, reaching the next more negative value −396.5 kJ at 1600 °C and is described by the following approximation equation ΔG_T_^o^ = −50.105T + 118.18 with the approximation confidence value (R^2^) equal to 0.9921.

Simulation of the transfer of Zn to gas with the simultaneous formation of the C_2_S compound according to Equation (8) and with the formation of the C_3_S compound according to Equation (9) with the simulation of the transition of Zn to gas is shown in Figure 2a. From the found values of the ΔG_T_° in Equations (8) and (9), it follows that they have the ability to flow and react in the entire temperature range of the investigations. Equations (8) and (9) have the following values of ΔG_T_° at 600 °C: −571.2 kJ and −518.7 kJ, respectively. Moreover, with a further increase in temperature, the values of ΔG_T_° −1442.3 kJ and −1534.4 kJ, respectively, at 1600 °C, begin to become more negative. This trend indicates their complete flow and reaction in the temperature range under study and is correspondingly described by the following approximation equations ΔG_T_° = −86.876T − 475.87 with an approximation confidence value (R^2^) equal to 0.9995 (for C_2_S) and ΔG_T_^o^ = −101.33T − 410.19, an approximation confidence value (R^2^) equal to 0.9997 (for C_3_S). At the same time, the values of ΔG_T_° of chemical Equations (8) and (9) at a temperature of 900 °C reach almost identical values and have the property of intersecting with more negative values of ΔG_T_° of Equation (9) in contrast to the values of Equation (8). In Figure 2a there is a distinct distortion of the curves of the values of ΔG_T_° of Equations (8) and (9) in the temperature range of 900–1200 °C, which is presumably in all probability a consequence of polymorphism of silica. Unlike Equations (4) and (5), Equations (8) and (9) have more negative values in the studied temperature range, in particular at 600 °C (by −521 and −516.4 kJ, respectively) and at 1600 °C (by −1101.8 and −1101.9 kJ, respectively); this is explained by the presence in the equations of zinc having a boiling point of 907 °C and its interaction during evaporation into the gas phase [7,8,32,35,39,41], and thereby contributes to the reduction in energy consumption, temperature, and intensification of these chemical equations.

The curve of ΔG_T_° values depending on the temperature of Equation (10) is shown in Figure 2b and has negative values, in particular −1012.8 kJ at 600 °C and −2096.2kJ at 1600 °C over the entire range of temperatures studied, which shows the possibility of complete flow and reaction in the presence of zinc ferrite with the formation of a mineral phase in the form of a compound: three calcium aluminate and the conversion of Zn to gas with the description of the following approximation equation ΔG_T_° = −108.94T − 892.05, with the approximation confidence value (R^2^) equal to 0.9997. The curve of the values of ΔG_T_^o^ as a function of the temperature of Equation (11) is shown in Figure 2b. Moreover, in comparison with Equation (10), chemical Equation (11) has the property of proceeding partially. Chemical Equation (11) has ΔG_T_^o^ in the temperature range from 600 to 1100 °C with positive values of 579.1 kJ and 11.8 kJ, respectively. The obtained positive values of the ΔG_T_^o^ Equation (11) demonstrate its impossibility of reacting and proceeding in the following temperature ranges: 600–1100 °C. However, in the process of further increase in temperature values, starting already at 1110.4 °C, the values of ΔG_T_° of Equation (11) begin to have negative values, in particular −0.08 kJ, which indicates the beginning of the reaction (8) in the presence of zinc ferrite with the formation of a phase of the mineralogical composition of the simulated sinter, clinker in the form of a compound, four calcium alumina ferrite, and the reaction of Zn in the form of gas. With a further increase in the temperature range of the studies, the ΔG_T_° of Equation (11) begins to have increasingly negative values, with the maximum value of −523 kJ reaching at 1600 °C and is described by the approximation equation ΔG_T_° = −112.35 T + 688.24, with the approximation accuracy value (R^2^) equal to 0.9994. Unlike chemical Equations (6) and (7), Equations (10) and (11) have both more negative and positive values in the temperature range under study, in particular at 600 °C (−1012.8 and 579.1 kJ, respectively) and at 1600 °C (−2096.2 and −523 kJ, respectively), this is explained by the presence of zinc in the equations [7,8,32,35,39,41], which contributes to the reduction in energy consumption, temperature, and intensification of the flow of these chemical equations. 

The dependences obtained by the results of the study and the results of replacing mineral raw materials with man-waste, reducing the firing temperature (100–200 K) and intensifying the processes of mineral formation of clinkers with simultaneous distillation of zinc into the gas phase with further capture, contribute to a significant reduction in the cost of cement clinker (≈25–30%) and complement the previously conducted studies of a number of scientists from various countries the world [42,43,44,45,46,47,48,49,50]. The authors of [50] did not investigate the effect of Zn or its compounds themselves, but only provided a review, noting that a number of compounds, including zinc, contribute to the mineralization, stabilization, and temperature of the process. The authors proposed the use of the NASA Aerospace Agency database, because thousands of variants of rocket fuel of various gases are modeled there, which in our subjective opinion has only an indirect relation of these processes to the processes of clinker formation. The authors, in the studies cited in the literature [50], considered the experience of calculating a number of interactions in the form of interaction of a raw mixture in its classical form, that is, using calcium oxides or silicon without the use of technological waste containing non-ferrous metals, such as zinc.

At the same time, prerequisites for the complex utilization of technogenic mineral waste from the enrichment of the mining and metallurgical industry as secondary raw materials for the chemical industry were created [51,52,53,54,55,56,57,58,59,60,61]. This is a new idea and has been experimentally tested in laboratory conditions and according to the results of research we obtained a patent for a utility model from the Republic of Kazakhstan, No. 6995 “Raw mix for the production of cement clinker” dated 8 April 2022, which confirms the novelty and protects our intellectual property.

## 5. Conclusions

Through thermodynamic simulation of environmental and population protection by utilization of technogenic tailings of enrichment, the following conclusions can be drawn:

− Technogenic mineral formations of the mining and metallurgical industry, containing Si, Al, and Fe oxides in their composition and being in a pulverized fraction are capable of harming human health and the environment, but at the same time act as a silicon-aluminum-iron-containing component for clinker charge in the following calculated optimal ratio of limestone and industrial waste in the raw mixture 80.9–82.5% and 17.5–19.1%, respectively;
− From the thermodynamic calculation of the formation of mineralogical phase formation of the sinter clinker simulation, it follows that in the first row of chemical Equations (4)–(7) at a temperature value of 1400 °C, depending on ΔG_T_^o^, the following sequence of clinker mineral formation is represented: Ca_3_SiO_5_ (−348.6 kJ); Ca_4_Al_2_Fe_2_O_10_ (−342.1 kJ); Ca_3_Al_2_O_6_ (−285 kJ); and Ca_2_SiO_4_ (−284.1 kJ);
− From the thermodynamic calculation of the formation of mineralogical phase formation of sinter clinker modeling, it follows that in the second series of chemical Equations (8)–(11) in the presence of zinc ferrite at a temperature value of 1400 °C, depending on ΔG_T_^o^, the following sequence of clinker mineral formation is represented: Ca_3_Al_2_O_6_ (−1874.1 kJ); Ca_3_SiO_5_ (−1319.3 kJ); Ca_2_SiO_4_ (−1254.8 kJ); and Ca_4_Al_2_Fe_2_O_10_ (−331.6 kJ);
− In chemical Equations (8)–(11) in the process of mineralogical phase formation of sinter clinker modeling in the presence of zinc ferrite, contributes, under equal conditions, to a decrease in temperature by 100–200 K and intensification of the flow of equations in comparison with known chemical Equations (4)–(7) with energy savings from 10.5 to 1589.1 kJ;
− In chemical Equations (8)–(11), in the process of mineralogical phase formation of sinter clinker modeling in the presence of zinc ferrite, Zn can be distilled as a gas at T = 1200–1400 K.

## Figures and Tables

**Table 1:** Gibbs energy dependence on temperature.

T, °C	Reaction Gibbs Energy, G, kJ			
	2CaCO_3_ + SiO_2_ → Ca_2_SiO_4_ + 2CO_2_	3CaCO_3_ + SiO_2_ → Ca_3_SiO_5_ + 3CO_2_	3CaCO_3_ + Al_2_O_3_ → Ca_3_Al_2_O_6_ + 3CO_2_	4CaCO_3_ + Al_2_O_3_ + Fe_2_O_3_ → Ca_4_Al_2_Fe_2_O_10_ + 4CO_2_
600	−571.2	−518.7	−1012.8	579.1
700	−650.2	−612.9	−1114	466.4
800	−731.2	−709	−1217.2	353.3
900	−825	−817.7	−1322.4	239.7
1000	−910.7	−918.1	−1429.5	125.9
1100	−998.2	−1020.1	−1538.2	11.8
1200	−1073.6	−1109.8	−1648.7	−102.5
1300	−1163.4	−1213.9	−1760.7	−216.9
1400	−1254.8	−1319.3	−1874.1	−331.6
1500	−1347.8	−1426.2	−1989.1	−446.4
1600	−1442.3	−1534.4	−2096.2	−523

## Data Availability

Data sharing not applicable.
